# Society Meetings

**Published:** 1906-12

**Authors:** 


### SOCIETY MEETINGS.

The Southern Surgical and Gynecological Association will hold its nineteenth annual meeting at the Hotel Belvidere, Baltimore, December 11, 12, and 13, 1906, under the presidency of Dr. George H. Noble, of Atlanta. Dr. Howard A. Kelly, of Baltimore, is chairman of the committee of arrangements and Dr. William D. Haggard of Nashville is secretary and one or the other of these, according to the nature of the communication, should be addressed concerning the meeting or the program.

---

The Medical Society of the County of Niagara held a regular meeting at Lockport, November 20, 1906, at which Dr. W. S. Renner of Buffalo read a paper on Diseases of the Ear. A board of censors was appointed which was instructed to employ an attorney to prosecute illegal practitioners within the jurisdiction of the society. The practice of contracting with lodges, societies, firms or corporations for professional services at reduced fees was condemned and any offender was made liable to expulsion.

---

The Medical Society of the State of New York will hold its one hundred and first annual meeting at Albany, Tuesday, Wednesday, and Thursday, January 29, 30, and 31, 1907, under the presidency of Dr. Joseph D. Bryant, of New York.

Dr. L. H. Neuman, 194 State Street, Albany, is chairman of the committee on scientific work, to whom all applications for places on the program should be addressed.

---

The Buffalo Academy of Medicine held meetings during the months of October and November, 1906, as follows:

*Section on Pathology.*—Tuesday, Oct. 30, at 8:30 P. M. Program: Newer conception of the nature and treatment of diabetes, Alfred C. Croftan, Chicago.

*Section on Surgery.*—Wednesday, November 7, at 8:30 P. M. Program: (a) Arthritis deformans of the spine, Prescott LeBreton; (b) The present status of lumbar anesthesia, Max C. Breuer; (c) Presentation of a patient showing the results of tendon transplantation in a case of anterior-poliomyelitis, R. O. Meisenbach.

*Section on Medicine.*—Tuesday, November 13, at 8:30 P. M. Program: A consideration of the pelvic articulations, Joel E. Goldthwait, Boston, President American Orthopedic Association; discussion opened by R. O. Meisenbach.

*Section on Pathology.*—Tuesday, November 20, at 8:30 P. M. Program: A lecture by Dr. Loeb of the University of Pennsylvania, illustrated with lantern slides.

*Section on Obstetrics and Gynecology.*—Tuesday, November 27, at 8:30 P. M. Program: (a) Obstetric lacerations, P. W. Van Peyma; (b) Management of occipital presentations, Earl P. Lothrop; (c) Reflex dyspepsias due to abdominal and pelvic diseases, Herman E. Hayd.

---

The Mississippi Valley Medical Association at its recent annual meeting held at Hot Springs, Ark., elected the following-named officers: president, H. Horace Grant, Louisville; first vice-president, G. A. Herbert, Hot Springs, Ark.; second vice-president, T. C. Witherspoon, Saint Louis; secretary, Henry E. Tuley, (reelected) Louisville; treasurer, S. O. Stanton (reelected) Chicago. The next annual meeting will be held at Columbus, in October, 1907.

---

The Association of Lehigh Valley Railway Surgeons at its recent annual meeting held in New York elected the following named officers: president, Grosvenor R. Trowbridge, Buffalo; secretary, J. C. Zern, Wilkesbarre, Pa. The next meeting will be held at Wilkesbarre.

THE Medical Society of the County of Wyoming at its recent meeting held at Castile in October, elected the following named officers: president, F. E. Bliss, Warsaw; vice-president, Mary Greene, Castile; secretary (reelected) B. H. Humphrey, Silver Springs.

